# Combined Foot and Eye Dominance Scale as a useful tool for the assessment of lateralization

**DOI:** 10.1192/j.eurpsy.2023.2258

**Published:** 2023-07-19

**Authors:** K. V. Akabalieva

**Affiliations:** Psychiatry, Medical University “Sofia”, Sofia, Bulgaria

## Abstract

**Introduction:**

Lateralization is the functional dominance of one of the dual organs of the body: eyes, arms, legs and even ears, during their spontaneous or purposeful actions. The handedness is influenced by many factors like geographic region, genetic and cultural factors as well as sex. The most used assessment for functional lateralization is hand dominance. When assessing foot and eye dominance, however, we find significantly higher left foot dominance as well as very strong left eye dominance in schizophrenic patients versus controls. We consider the explanation of this results is because in Bulgaria, during the communist regime (before 1990) left hand writing was under cultural pressure. Also handedness itself is influenced by other cultural factors as mentioned already. All of the above made us conclude that foot and eye dominance may assess much better the lateralization than hand dominance.

**Objectives:**

The aim of the study was to investigate the relaibility (internal consistency) of a combined Foot and Eye Dominance Scale in women- patients with schizophrenia and healthy subjects.

**Methods:**

A sample of 94 women- scizophrenia patients and healthy controls were assessed with a Combined afaoot and Eye Dominance Scale. It consisted of two subscales Foot Dominance Subscale and Eye Dominance Subscale. The Foot Dominance Subscale included a modified Chapman & Chapman Foot Dominance Scale and a new Complex Task Scales with four foot tests, reflecting on complex tasks. The Eye Dominance Subscale included three eye tests. Scale reliability statistics (item-scale statistics, summary statistics for the items, Cronbach’s alpha), non-parametric Mann-Whitney test and Spearman’s rank correlation coefficient were used.

**Results:**

Considerable differences were found in the contribution of the single items to the Combined Foot and Eye Dominance Scale. Some items show greater means than other items (from 0,14 to 0,80), which suggested greater phenogenetic component and consequently greater contribution of these items to the total scale mean. The mean correlation between the items of the Combined Foot and Eye Dominance Scale was positive (0,32), indicating good internal consistency of the scale.

**Conclusions:**

The Combined Foot and Eye Dominance Scale more strongly and objectively reflect leftedness and could be a more useful tool for the assessment of lateralization irrespectively of culture and nation. The Combined Scale allows cross-cultural worldwide equivalence to the different studies in different neuroontogenetic diagnoses with presumed abnormal cerebral asymmetry.

**Disclosure of Interest:**

None Declared

